# Scientometrics and academia

**DOI:** 10.17305/bb.2023.10173

**Published:** 2024-04-01

**Authors:** Enver Zerem, Semir Vranić, Kemal Hanjalić, Dejan B Milošević

**Affiliations:** 1 Department of Medical Sciences, Academy of Sciences and Arts of Bosnia and Herzegovina, Sarajevo, Bosnia and Herzegovina; 2 College of Medicine, QU Health, Qatar University, Doha, Qatar; 3 Department of Technical Sciences, Academy of Sciences and Arts of Bosnia and Herzegovina, Sarajevo, Bosnia and Herzegovina; 4 University of Sarajevo, Sarajevo, Bosnia and Herzegovina; 5 Department of Natural and Mathematical Sciences, Academy of Sciences and Arts of Bosnia and Herzegovina, Sarajevo, Bosnia and Herzegovina; 6 Faculty of Science, University of Sarajevo, Sarajevo, Bosnia and Herzegovina

**“Count what is countable, measure what is measurable and what is not measurable, make measurable” (G. Galileo)**

## Scientometrics and academia

The social significance and quality of every human activity are proportional to its usefulness to the social community. Science belongs to the very top of the processes and events in the history of humankind that strongly influenced the development of society, which over time transformed it and contributed to the common good. Science produced new knowledge that made it possible for billions of people to rise out of poverty, develop industrialization and mass communication, eradicate many dangerous diseases for humankind, and enable humans to leave their footprints on the moon. Science is a human activity that produces new knowledge presented through innovations, patents, and publications, aimed at solving the problems facing humanity [1–3].

The academic community, represented by higher education institutions that have the most resources and abilities to create science, is inevitably brought to the center of attention when it comes to the development of science and its quality. Simultaneously, higher education institutions have another very important task, namely the education of new generations of scientists and experts who should continue the development and improvement of science and society [1–3]. The task of successfully implementing such important and complex activities, as science and education, imposes the necessity for constant monitoring of the quality of the work of higher education institutions, both in scientific and educational activities. However, it is very difficult to apply the right criteria that can objectively value the scientific and even more difficult the educational segment. Although both activities are equally important, scientific research presented through innovations, patents, and publications can be evaluated more precisely compared with educational and other activities of academic institutions, inevitably imposing itself as a dominant measure in the evaluation of the quality of higher education as a whole [3–5].

The basic product of scientific research is information published in scientific journals, which are the most important means of spreading knowledge and the most frequently used criteria for academic and scientific evaluation, as well as for the distribution of funds for scientific research. The fact is that there is a wide range of other scientific and teaching activities (patents, research project grants, leadership in national or international academic societies, membership in editorial boards of respected journals, mentorship of doctoral dissertations, teaching skills, appearances in the media, articles in the lay press and the like) that reflect the scientific credibility of scientists as well as the academic credibility of the university teachers [6–8].

However, the relevant science metrics systems (SMSs) evaluate only publications, without considering other criteria mentioned above, which undoubtedly have importance in scientific and academic values. The most important reason for this is the fact that scientometrics, as a measure of scientific achievements, seeks to follow the logic of science as a universal value and to create SMSs that will evaluate all scientific disciplines and all scientific production uniquely. In contrast, the mentioned academic activities are very heterogeneous, with specific characteristics that would require very different parameters for evaluation, making it hard to evaluate them according to unique criteria [3, 7].

Nevertheless, frequent criticism of the scientometrics, due to its focus only on citation score, which is blind to other contents and merits pertinent to the scientific and academic credibility of scientists, is not justified nor can it be an excuse for not applying SMSs in academic and scientific valorizations. Although the mentioned activities cannot be universally evaluated, academic and research funding institutions can include them along with scientometrics in the evaluation if they consider that they are essential for the specific ranking [3, 7, 8]. One often gets the impression that the impossibility of including other teaching and scientific activities (except citation) in SMSs is just an excuse for not applying scientometrics in academic and scientific valorization.

The fact is that SMSs are often criticized both by respected scientists and those who are not relevant according to those criteria. Even Eugene Garfield himself, the originator of the introduction of citations in the evaluation of scientific articles [9], pointed out some of the shortcomings of citations, highlighting as illogical the example of the biochemist Oliver H. Lowry whose article on the method of determining protein in liquid, was cited more times than the total number of quotes from any Nobel laureate [10]. As an example of justified criticism of some of the well-known and generally accepted SMSs such as H-index and number of citations, is the fact that some scientists have hundreds or thousands of citations and high H-indexes, but have a meager number of citations in the articles in which they are first or senior/corresponding authors. A particular disadvantage for the objectivity of SMSs is the valorization of so-called “position papers”, in which certain scientific and professional associations or pharmaceutical companies express their consensuses or positions on some issues, in which hundreds or even thousands of authors are listed and receive the same (total) number of citations of that article, although it is known that only a small number of authors participate in writing such articles [7]. The very existence of a large number of SMSs is convincing evidence that there is no perfect SMS that can accurately measure the scientific relevance of scientists and scientific journals.

However, despite all the weaknesses, it is an indisputable fact that scientometrics, as a system for evaluating the citation of scientific articles, is the most relevant measure of the value of a scientific article, which can be universally applied. It should be pointed out that the greatest weakness of scientometrics is rooted in its greatest virtue: the effort to achieve universal criteria that will uniquely evaluate all scientific disciplines and all regions around the world, regardless of the level of development of individual countries and regions. It is obvious that harmonizing universal criteria with the specificities of certain scientific fields, evaluation of different types of articles, non-unique criteria for the order of authors in a scientific article, as well as the specificities of the work of scientists working in less developed compared to highly developed scientific centers and countries encounters serious difficulties [7, 11].

In the last ten years, several new SMSs have been published to overcome the aforementioned discrepancies and illogicalities, but with an effort to maintain the universality of the criteria [7, 12–16]. Since 2018, a group of scientists from Stanford University has evaluated the scientific contributions of scientists based on the impact of citations and calculated the top 2% in each scientific discipline and sub-discipline. They have created “a publicly available database of 100,000 top scientists that provides standardized information on citations, h-index, co-authorship adjusted hm-index, citations to papers in different authorship positions, and a composite indicator”… “Scientists are classified into 22 scientific fields and 176 subfields. Field and subfield specific percentiles are also provided for all scientists who have published at least five papers” [17, 18]. In this evaluation, they used standard scientometric parameters, but they also added some specific criteria that were important to eliminate the shortcomings in their ranking. We think, that this is a good recipe for the practical application of SMSs in the evaluation of specific disciplines or the specific purpose of the evaluation, which will be carried out following the internationally accepted criteria, but will respect the specificities of a certain scientific discipline or the purpose of the evaluation.

It is an indisputable fact that the existing SMSs have multiple shortcomings and are not optimal for the objective assessment of the quality of scientific research and the importance and merits of scientists. That is why all purposeful suggestions for their improvement are much desired. However, we must not equate proposals to improve SMSs with the refusal to implement internationally recognized criteria due to their alleged imperfection. The valorization of scientific research and academic progress, without coordination with internationally recognized scientific criteria, would lead to the undeniable risk that decision-makers in the academic community, deciding subjectively, would lower the criteria threshold below the level of relevant international standards and significantly devalue the valorization process. The subjective valorization of scientific research and academic advancement would be especially risky if it were applied in small and underdeveloped academic communities with poor scientific infrastructure [3, 5, 19].

It is interesting that in academic communities, everyone agrees that science is a universal concept and that there is no “local” science. But when it comes to the valorization of science according to internationally recognized criteria, disagreements arise. However, it is an indisputable fact that if there is no “local” science, there are no local criteria for its valorization, which do not follow the international scientific criteria. Besides, although the modern SMS criteria suffer from numerous shortcomings and are faced with the opposition of a part of the academic community, they are increasingly becoming ‘the fact of life’, a part of the reality and the prerequisite of the existence of thousands of academics and their institutions around the world. Scientometrics will continue to exist and develop, as a science in itself, not because it is the ideal criterion for evaluating science, but because “measurement” is the essence of science and scientometrics is the only universal measure for scientific evaluation, which probably will not have an adequate alternative in the foreseeable future.
